# Supplementary material for: Associations of parental depression during adolescence with cognitive development in later life in China: A population-based cohort study
Source: PLoS Med. 2021 Jan 11;18(1):e1003464. doi: 10.1371/journal.pmed.1003464 (PMC7799791; doi:10.1371/journal.pmed.1003464)
Supplement: S1 STROBE Checklist — STROBE, Strengthening the Reporting of Observational Studies in Epidemiology. (DOC) [file pmed.1003464.s001.doc]

STROBE Statement—Checklist of items that should be included in reports of ***cohort studies***

|  | Item No | Recommendation | Response |
| --- | --- | --- | --- |
| **Title and abstract** | 1 | (*a*) Indicate the study’s design with a commonly used term in the title or the abstract | The study’s design is indicated in the manuscript title. |
|  |  | (*b*) Provide in the abstract an informative and balanced summary of what was done and what was found | Such content is provided in the Methods and Findings section of the abstract. |
| Introduction | | |  |
| Background/rationale | 2 | Explain the scientific background and rationale for the investigation being reported | The scientific background and rationale for the study are described in the first three paragraphs of the Introduction. |
| Objectives | 3 | State specific objectives, including any pre-specified hypotheses | The objectives of the study are described in the fourth paragraph of the Introduction. |
| Methods | | |  |
| Study design | 4 | Present key elements of study design early in the paper | *These elements are presented in the Methods section. Including, for example****, “Data used in this study were from the China Family Panel Studies (CFPS). CFPS is a nationally representative, longitudinal survey conducted by the Institute of Social Science Survey at Peking University in collaboration with the Survey Research Center at the University of Michigan [13].”*** and ***“To assess the impact of parental depression on offspring cognitive development, we set up the data in a panel form with parents’ depression status measured in 2012 and offspring cognitive performance measured in 2014, 2016, and 2018. Among the 2,281 offspring identified in 2012, we successfully followed up 85.9% of the offspring in 2014, 72.1% in 2016, and 63.7% in 2018 (Table 1).”*** |
| Setting | 5 | Describe the setting, locations, and relevant dates, including periods of recruitment, exposure, follow-up, and data collection | The setting, locations, relevant dates, and periods of recruitment are reported in the “Data source and study sample” section of the Methods (***e.g., “CFPS was first launched in 2010 with subsequent rounds of data collected in 2012, 2014, 2016, and 2018. The target sample of CFPS consists of around 16,000 Chinese households, representing 95% of the Chinese population [13].”***. Exposure is described in the “Exposure measure” section of the Methods: “***Parental depression status was measured using a 20-item full version of the Center for Epidemiologic Studies Depression Scale (******CES-D 20) in the 2012 wave.***” |
| Participants | 6 | (*a*) Give the eligibility criteria, and the sources and methods of selection of participants. Describe methods of follow-up | The eligibility criteria, and the sources and methods of selection of participants are provided in the “Data source and study sample” section of the Methods: ***“Our study used the 2012 survey as the baseline when depression measurement for at least one parent was available. Of all the households included in the CFPS data, there were 2,406 offspring aged 10-15 years in the CFPS 2012 survey. Among them, we excluded 135 offspring with both parents’ depression measures marked as ‘unknown,’ ‘refuse to answer,’ or ‘inapplicable’. Thus, the final sample comprised 2,281 offspring.”*** |
|  |  | (*b*) For matched studies, give matching criteria and number of exposed and unexposed | N/A |
| Variables | 7 | Clearly define all outcomes, exposures, predictors, potential confounders, and effect modifiers. Give diagnostic criteria, if applicable | We have three separate sections in Methods to describe the exposure measure, outcome measures, and Covariates, respectively.  The exposure measure is defined in “Exposure measure” section: “***Parental depression status was measured using a 20-item full version of the Center for Epidemiologic Studies Depression Scale (CES-D 20) in the 2012 wave...***”  The outcome measures are defined in “Outcome measures” section: “***The CFPS has developed two sets of tests to measure the cognitive performance of individuals aged 10 years or older. Cognitive performance was defined as the composition of “crystalized intelligence” and “fluid intelligence”, which were assessed using different measures in different years [23,24]. Specifically, in the waves of 2010, 2014, and 2018, mathematics and vocabulary tests were conducted to assess “crystallized intelligence”, referred to as the ability to use knowledge acquired through past learning, experience, and education [23]. Test items were drawn from the standard curriculums in Chinese primary and secondary schools; in the waves of 2012 and 2016, immediate word-recall, delayed word-recall, and number series tests were collected to measure “fluid intelligence”, referred to as the ability to reason and solve problems in unique and novel situations [23,24].***”  The confounders are defined in “*Covariates” section:* ***“Following previous practices [10,27], we adopted covariates at offspring-, parents- and household-levels (see Table 1). Offspring characteristics included age, sex, and birth order. Parents’ characteristics included parents’ education levels, age, whether living together with the offspring for more than eight months in the past 12 months, and employment status. Except for parents’ education level and ages, all other parents’ characteristics were dichotomous variables. Household characteristics included place of residence (i.e., urban or rural), household income in log scale, and the number of offspring in the household.”*** |
| Data sources/ measurement | 8* | For each variable of interest, give sources of data and details of methods of assessment (measurement). Describe comparability of assessment methods if there is more than one group | Such information is provided in the “Data source and study sample” section of the Methods, e.g., “***Data used in this study were from the China Family Panel Studies (CFPS).***” And “***In CFPS 2012, parents rated the 20 symptom items over the past week (e.g., I feel lonely) on a 4-point scale of 0 to 3, which were ‘almost never (less than 1 day)’, ‘sometimes or occasionally (1 or 2 days a week)’, ‘frequently (3 or 4 days a week)’, and ‘most of the time (5 days or more a week)’.*** And ***“Test items were drawn from the standard curriculums in Chinese primary and secondary schools.”*** |
| Bias | 9 | Describe any efforts to address potential sources of bias | As described in the Methods section, to deal with the potential attrition issues, we conducted multiple imputation to impute for observations loss of follow-up or for observations with key covariates missing in 2014, 2016, and 2018. Besides this, we conducted a series of sensitivity analyses, controlling for offspring cognitive performance during toddlerhood (i.e., age to speak a whole sentence, and age to count from 1 to 10); offspring cognitive performance in 2012 when parental depression was assessed; offspring CES-D score measured in 2012; parental cognitive scores measured in 2010. |
| Study size | 10 | Explain how the study size was arrived at | This is described in the “Data source and study sample” section of the Methods, specifically, “***Of all the households included in the CFPS data, there were 2,406 offspring aged 10-15 years in the CFPS 2012 survey. Among them, we excluded 135 offspring with both parents’ depression measures marked as “unknown”, “refuse to answer”, or “inapplicable”. Thus, the final sample comprised 2,281 offspring***.” |
| Quantitative variables | 11 | Explain how quantitative variables were handled in the analyses. If applicable, describe which groupings were chosen and why | This is described in the “Statistical analysis” section of the Methods, see above in the response to item 7. |
| Statistical methods | 12 | (*a*) Describe all statistical methods, including those used to control for confounding | The statistical methods used are thoroughly described in the “Statistical analyses” section of the Methods. |
|  |  | (*b*) Describe any methods used to examine subgroups and interactions | This is described at the end of “Statistical analyses” section of the Methods, for example, ***“… we stratified the sample by the severity of parental depression and constructed linear regression models (controlling for all*** ***offspring, parents, and household characteristics) to examine the association between severity of depression and offspring cognitive development.”*** |
|  |  | (*c*) Explain how missing data were addressed | We conducted sensitivity analysis using multiple imputation to impute for observations loss of follow-up or for observations with key covariates missing in 2014, 2016, and 2018. |
|  |  | (*d*) If applicable, explain how loss to follow-up was addressed | Among the 2,281 offspring identified in the 2012 survey, 1,960 (85.9%), 1,644 (72.1%), and 1,452 (63.7%) were followed-up in 2014, 2016, and 2018, respectively. Thus, our first follow-up had a very respectable completion rate, though this did fall with subsequent rounds (as might be expected). Following the widely adopted criteria in previous studies, we determined that attrition bias in the current study is likely to be small because there were no systematic differences in baseline offspring, parents, and household characteristics between participants followed-up and those who were lost.  Furthermore, we conducted a sensitivity analysis to address the attrition issue. Following the approach in earlier studies, we used multiple imputation to impute for observations loss of follow-up or for observations with key covariates missing in 2014, 2016, and 2018. In our imputation, we included all the variables on offspring characteristics, parents’ characteristics, household characteristics, as well as parental CES-D scores. |
|  |  | (*e*) Describe any sensitivity analyses | Sensitivity analyses are described in the last paragraph of the “Statistical analyses” section of the Methods, e.g., ***“We also conducted six sets of sensitivity analyses to assess the robustness of our results. We added respectively 1) offspring retrospective cognitive measures during their toddlerhood…”*** |
| Results | | |  |
| Participants | 13* | (a) Report numbers of individuals at each stage of study—eg numbers potentially eligible, examined for eligibility, confirmed eligible, included in the study, completing follow-up, and analysed | Same as the response to item 10. |
|  |  | (b) Give reasons for non-participation at each stage | NA |
|  |  | (c) Consider use of a flow diagram | - |
| Descriptive data | 14* | (a) Give characteristics of study participants (eg demographic, clinical, social) and information on exposures and potential confounders | Characteristics of study participants are presented in Table 1 and S3 Table, and in the first paragraph of the Results section: e.g., “***Table 1 shows attrition status and summary statistics for key characteristics of the study sample (n=2,281). Among the 2,281 offspring identified in the 2012 survey, 1,960 (85.9%), 1,644 (72.1%), and 1,452 (63.7%) were followed-up in 2014, 2016, and 2018, respectively. There was no significant difference for most baseline parental characteristics between those offspring who were followed up and those who were lost with the exception of maternal education and whether either parent lived with the offspring for more than eight months in the past 12 months.”*** |
|  |  | (b) Indicate number of participants with missing data for each variable of interest | In our study sample, the only variable with missing value is household income. We have marked the number of missingness in the footnote of S3 Table: “***There were 55 of the 2,281 children with household income per capita missing, including 36 from the non-depression group and 19 from the depression group.***” |
|  |  | (c) Summarise follow-up time (eg, average and total amount) | Follow-up time is summarised in the first paragraph of the Result: “***Among the 2,281 offspring identified in the 2012 survey, 1,960 (85.9%), 1,644 (72.1%), and 1,452 (63.7%) were followed-up in 2014, 2016, and 2018, respectively.”*** |
| Outcome data | 15* | Report numbers of outcome events or summary measures over time | The numbers of outcome events are presented in the text, all figures and Table 3. |
| Main results | 16 | (*a*) Give unadjusted estimates and, if applicable, confounder-adjusted estimates and their precision (eg, 95% confidence interval). Make clear which confounders were adjusted for and why they were included | Adjusted, unadjusted and 95% confidence intervals are provided and covariates were mentioned in the footnotes of available tables and figures. |
|  |  | (*b*) Report category boundaries when continuous variables were categorized | NA |
|  |  | (*c*) If relevant, consider translating estimates of relative risk into absolute risk for a meaningful time period | NA |
| Other analyses | 17 | Report other analyses done—eg analyses of subgroups and interactions, and sensitivity analyses | We conducted three effect modification analyses: 1. Effect modification by offspring age at exposure, 2. Effect modification by offspring gender, 3. Stratified the sample by the severity of parental depression. The results are reported in Figs 2-7, and S5 Table. |
| Discussion | | |  |
| Key results | 18 | Summarise key results with reference to study objectives | The key results are summarised in the first paragraph of the Discussion, e.g., “***Using a population-based cohort in China, we found that both maternal depression and paternal depression when the offspring were 10 to 15 years were associated with worse cognitive performance up to six years later…”*** |
| Limitations | 19 | Discuss limitations of the study, taking into account sources of potential bias or imprecision. Discuss both direction and magnitude of any potential bias | Limitations of the study are described in the Discussion, e.g., “***Several limitations of our study should be noted. First, we used self-rated depression symptoms as the exposure measure. However, this approach is the most commonly adopted one in epidemiological studies and considered to be more scientifically sound than a binary diagnostic approach [17,18].***..” |
| Interpretation | 20 | Give a cautious overall interpretation of results considering objectives, limitations, multiplicity of analyses, results from similar studies, and other relevant evidence | A cautious overall conclusion has been given in the last two paragraphs of the Discussion section. |
| Generalisability | 21 | Discuss the generalisability (external validity) of the study results | The generalisability of the study results to the Chinese population as a whole and to non-Chinese populations is discussed in the last paragraph of the Discussion section. |
| Other information | | |  |
| Funding | 22 | Give the source of funding and the role of the funders for the present study and, if applicable, for the original study on which the present article is based | The funding information is provided. |

*Give information separately for exposed and unexposed groups.

**Note:** An Explanation and Elaboration article discusses each checklist item and gives methodological background and published examples of transparent reporting. The STROBE checklist is best used in conjunction with this article (freely available on the Web sites of PLoS Medicine at http://www.plosmedicine.org/, Annals of Internal Medicine at http://www.annals.org/, and Epidemiology at http://www.epidem.com/). Information on the STROBE Initiative is available at http://www.strobe-statement.org.
