# Supplementary material for: Associations of parental depression during adolescence with cognitive development in later life in China: A population-based cohort study
Source: PLoS Med. 2021 Jan 11;18(1):e1003464. doi: 10.1371/journal.pmed.1003464 (PMC7799791; doi:10.1371/journal.pmed.1003464)
Supplement: S1 Table — (DOCX) [file pmed.1003464.s002.docx]

**S1 Table. Measurement of depression symptoms**

| **Survey year** | **Recall period** | **Questions** | **Scores for the answers** | **Total score range** | **Categories** |
| --- | --- | --- | --- | --- | --- |
| 2012 | In the past week | I am annoyed by some trifles. | 0: Almost never (less than one day) 1: Sometimes (1-2 days) 2: Often (3-4 days) 3: Most of the time (5-7 days) | 0-60 | 0-15: No depression symptoms 16-24: Moderate depression symptoms 25-60: Severe depression symptoms[1–4] |
|  |  | I don't want to east and have a poor appetite. |  |  |  |
|  |  | I feel depressed even though I receive help from my family and friends. |  |  |  |
|  |  | I find it hard to focus on what I am doing. |  |  |  |
|  |  | I am depressed. |  |  |  |
|  |  | I find it difficult to do anything. |  |  |  |
|  |  | I feel that I have been a loser all the time. |  |  |  |
|  |  | I feel scared. |  |  |  |
|  |  | I have a poor sleep. |  |  |  |
|  |  | I talk less than usual. |  |  |  |
|  |  | I feel lonely. |  |  |  |
|  |  | I find people are unfriendly to me. |  |  |  |
|  |  | I have cried or want to cry. |  |  |  |
|  |  | I feel sad. |  |  |  |
|  |  | I feel other dislike me. |  |  |  |
|  |  | I feel that I am unable to keep on with my life. |  |  |  |
|  |  | I feel that I'm better than someone else. | 0: Most of the time (5-7 days) 1: Often (3-4 days) 2: Sometimes (1-2 days) 3: Almost never (less than one day) |  |  |
|  |  | I am hopeful about the future. |  |  |  |
|  |  | I am happy. |  |  |  |
|  |  | I have a happy life. |  |  |  |
|  |  | Feel nervous |  |  |  |
|  |  | Feel upset and cannot remain calm |  |  |  |
|  |  | Feel hopeless about the future |  |  |  |
|  |  | Feel that everything is difficult |  |  |  |
|  |  | Think life is meaningless |  |  |  |

**Reference:**

1. Radloff LS. The CES-D Scale: A Self-Report Depression Scale for Research in the General Population. Appl Psychol Meas. 1977;

2. Weissman MM, Sholomskas D, Pottenger M, Prusoff BA, Locke BZ. Assessing depressive symptoms in five psychiatric populations: A validation study. Am J Epidemiol. 1977;

3. Li Z, Hicks MHR. The CES-D in Chinese American women: Construct validity, diagnostic validity for major depression, and cultural response bias. Psychiatry Res. 2010;

4. Stahl D, Sum CF, Lum SS, Liow PH, Chan YH, Verma S, et al. Screening for Depressive Symptoms: Validation of the Center for Epidemiologic Studies Depression Scale (CES-D) in a Multiethnic Group of Patients With Diabetes in Singapore. Diabetes Care. 2008;
