# Supplementary material for: Associations of parental depression during adolescence with cognitive development in later life in China: A population-based cohort study
Source: PLoS Med. 2021 Jan 11;18(1):e1003464. doi: 10.1371/journal.pmed.1003464 (PMC7799791; doi:10.1371/journal.pmed.1003464)
Supplement: S2 Table — (DOCX) [file pmed.1003464.s003.docx]

**S2 Table. Design of the cognitive tests**

| **Test** | **Year of collection** | **Test design and score range** | **Cognitive ability tested** | **Previous literatures** |
| --- | --- | --- | --- | --- |
| Mathematics | 2010, 2014, 2018 | **Test design:** The respondents are asked to answer 24 mathematical questions sorted according to the difficulty level (from the easiest to the hardest). The test is terminated upon three wrong responses in sequence. The final score is the rank of the hardest question that the respondent answers correctly.  **Score range**: 0 (lowest) to 24 (highest) | Mathematical ability, measuring the ability to obtain, process, and retain mathematical information[1] | [2-5] |
| Vocabulary | 2010, 2014, 2018 | **Test design:** The respondents are asked to answer 34 vocabulary recognition questions sorted according to the difficulty level (from the easiest to the hardest). The test is terminated upon three wrong responses in sequence. The final score is the rank of the hardest question that the respondent answers correctly.  **Score range**: 0 (lowest) to 34 (highest) | Verbal ability, measuring school-related skills including word knowledge and verbal concept formation [6] | [2–4,7] |
| Immediate word recall | 2012, 2016 | **Test design:** Interviewers read a randomly selected list of 10 simple nouns (e.g., doctor, rice, river, etc.) to the respondents and asked them to recall the words immediately after the reading.  **Score range**: 0 (lowest) to 10 (highest) | Working memory capacity, measuring the ability to store, focus attention on and manipulate information for a relatively short period of time (usually a few seconds) [8] | [4,9–11] |
| Delayed word recall | 2012, 2016 | **Test design:** Interviewers read a randomly selected list of 10 simple nouns (e.g., doctor, rice, river, etc.) to the respondents and asked them to recall the words five minutes after the reading.  **Score range**: 0 (lowest) to 10 (highest) | Short-terms memory function, usually used to detect memory impairment [12] | [4,9–11] |
| Number series | 2012, 2016 | **Test design:** Number series test is a two-stage adaptive test requiring respondents to fill in the missing number to fit the numerical pattern: in the first stage, all respondents were asked the same set of questions with three items (an easier item, a moderately difficult item, and a more difficult item) and were scored 0 to 3 according to the number of correct items; in the second stage, each respondent was asked one of the four sets, each with three items, according to the scores they got in the first stage.  **Score range**: 0 (lowest) to 15 (highest) | Numerical reasoning ability, measuring the ability to interpret, analyze, and draw logical conclusions based on the data provided [13] | [4,9,10,14] |

**Reference:**

1. Saß S, Kampa N, Köller O. The interplay of g and mathematical abilities in large-scale assessments across grades. Intelligence. 2017;

2. Chen X, Zhang X, Zhang X. Smog in Our Brains: Gender Differences in the Impact of Exposure to Air Pollution on Cognitive Performance. IZA Discuss Pap No 10628 [Internet]. [cited 2017 Apr 6]; Available from: https://papers.ssrn.com/sol3/papers.cfm?abstract_id=2940618

3. Li Z, Chen L, Li M, Cohen J. Prenatal exposure to sand and dust storms and children’s cognitive function in China: a quasi-experimental study. Lancet Planet Heal. 2018;2(5).

4. Huang G, Xie Y, Xu H. Cognitive Ability: Social Correlates and Consequences in Contemporary China. Chin Sociol Rev. 2015;47(4):287–313.

5. Ojose O. Applying Piaget’s Theory of Cognitive Development to Mathematics Instruction. Math Educ. 2008;

6. Marshalek B. Trait and process aspects of vocabulary knowledge and verbal ability. 1981.

7. Kate N, Snowling MJ. Semantic Processing and the Development of Word-Recognition Skills: Evidence from Children with Reading Comprehension Difficulties. J Mem Lang. 1998 Jul 1;39(1):85–101.

8. Wilhelm O, Hildebrandt A, Oberauer K. What is working memory capacity, and how can we measure it? Front Psychol. 2013;

9. Yeung WJJ, Gu X. Left Behind by Parents in China: Internal Migration and Adolescents’ Well-Being. Marriage Fam Rev. 2016;

10. Reeve CL, Heeney MD, Woodley of Menie MA. A systematic review of the state of literature relating parental general cognitive ability and number of offspring. Pers Individ Dif. 2018;

11. McArdle JJ, Fisher GG, Kadlec KM. Latent Variable Analyses of Age Trends of Cognition in the Health and Retirement Study, 1992-2004. Psychol Aging. 2007;

12. Zhao Q, Lv Y, Zhou Y, Hong Z, Guo Q. Short-Term Delayed Recall of Auditory Verbal Learning Test Is Equivalent to Long-Term Delayed Recall for Identifying Amnestic Mild Cognitive Impairment. PLoS One. 2012;

13. Süß HM, Oberauer K, Wittmann WW, Wilhelm O, Schulze R. Working-memory capacity explains reasoning ability - And a little bit more. Intelligence. 2002;

14. Wise DA, McArdle JJ, Smith JP, Willis R. Cognition and Economic Outcomes in the Health and Retirement Survey. In: Explorations in the Economics of Aging. 2013.
