# Supplementary material for: Associations of parental depression during adolescence with cognitive development in later life in China: A population-based cohort study
Source: PLoS Med. 2021 Jan 11;18(1):e1003464. doi: 10.1371/journal.pmed.1003464 (PMC7799791; doi:10.1371/journal.pmed.1003464)
Supplement: S3 Table — (DOCX) [file pmed.1003464.s004.docx]

**S3 Table. Summary table of the sample’s characteristics at the baseline (2012)**

|  | **Total** | **Non-depression group (Neither parent had depression symptoms)** | **Depression group (****One or both parents had depression symptoms)** | **P value^1^** |
| --- | --- | --- | --- | --- |
| **No. of observations aged 10-15 in 2012** | **2,281** | **1,426** | **855** | **NA** |
| **Offspring characteristics** |  |  |  |  |
| Child age in 2012^#^ | 12.60 (0.04) | 12.59 (0.05) | 12.62 (0.06) | 0.624 |
| Child sex is female | 1,088 (47.7%) | 696 (48.8%) | 392 (45.9%) | 0.171 |
| Birth order in 2012 |  |  |  |  |
| 1^st^ | 1,626 (71.3%) | 1,033 (72.4%) | 593 (69.4%) | 0.003 |
| 2^nd^ | 545 (23.9%) | 341 (23.9%) | 204 (23.9%) |  |
| 3^rd^ or more | 110 (4.8%) | 52 (3.7%) | 58 (6.8%) |  |
| **Parents' characteristics** |  |  |  |  |
| Maternal education level in 2012 | |  |  |  |
| ≤6 years | 1,194 (52.4%) | 684 (48.0%) | 510 (59.7%) | <0.001 |
| 7-9 years | 540 (23.7%) | 356 (25.0%) | 184 (21.5%) |  |
| ≥10 years | 547 (24.0%) | 386 (27.1%) | 161 (18.8%) |  |
| Paternal education level in 2012 | |  |  |  |
| ≤6 years | 826 (36.2%) | 428 (30.0%) | 398 (46.6%) | <0.001 |
| 7-9 years | 639 (28.0%) | 439 (30.8%) | 200 (23.4%) |  |
| ≥10 years | 816 (35.8%) | 559 (39.2%) | 257 (30.1%) |  |
| Maternal age in 2012 |  |  |  |  |
| ≤35 | 523 (22.9%) | 332 (23.3%) | 191 (22.3%) | 0.914 |
| 36-40 | 940 (41.2%) | 589 (41.3%) | 351 (41.1%) |  |
| 41-45 | 593 (26.0%) | 368 (25.8%) | 225 (26.3%) |  |
| ≥46 | 225 (9.9%) | 137 (9.6%) | 88 (10.3%) |  |
| Paternal age in 2012 |  |  |  |  |
| ≤35 | 286 (12.5%) | 189 (13.3%) | 97 (11.4%) | 0.296 |
| 36-40 | 915 (40.1%) | 568 (39.8%) | 347 (40.6%) |  |
| 41-45 | 690 (30.3%) | 438 (30.7%) | 252 (29.5%) |  |
| ≥46 | 390 (17.1%) | 231 (16.2%) | 159 (18.6%) |  |
| Offspring lived together with the mother for more than eight months in the past 12 months, 2012 | 1,618 (70.9%) | 998 (70.0%) | 620 (72.5%) | 0.198 |
| Offspring lived together with the father for more than eight months in the past 12 months, 2012 | 1,378 (60.4%) | 844 (59.2%) | 534 (62.5%) | 0.122 |
| Mother was employed or self-employed in 2012 | 1,893 (83.0%) | 1,184 (83.0%) | 729 (85.3%) | 0.055 |
| Father was employed or self-employed in 2012 | 2,018 (88.5%) | 1,241 (87.0%) | 757 (88.5%) | 0.090 |
| **Household characteristics** |  |  |  |  |
| Lived in rural areas in 2012 | 1,717 (75.3%) | 1,035 (72.6%) | 682 (79.8%) | <0.001 |
| Household income per capita in 2012 (¥ USD), log scale^#^ | 6.81 (0.03) | 6.95 (0.03) | 6.57 (0.04) | <0.001 |
| Number of children in the household in 2012^#^ | 2.08 (0.02) | 2.01 (0.02) | 2.27 (1.10) | <0.001 |
| **Offspring earlier cognitive ability for supplementary analysis**^2^ | |  |  |  |
| Age to speak a complete sentence in month (N=1,426) ^#^ | 24.68 (0.19) | 24.41 (0.24) | 25.08 (0.30) | 0.085 |
| Age to count from 1 to 10 in month (N=1,386) ^#^ | 35.19 (0.25) | 34.39 (0.30) | 35.49 (0.45) | 0.020 |

**Note:**

1. P values were generated from t-test for dichotomous variables (e.g., child sex) and continuous variables (e.g., child age), and from Chi-squared test for categorical variables (e.g. maternal education);

2. Data on offspring’s earlier cognitive ability were from the survey conducted in 2010;

# We placed standard errors in the parenthesis following the mean values for these items.

3. There were 55 of the 2,281 children with household income per capita missing, including 36 from the non-depression group and 19 from the depression group.
