# Supplementary material for: Associations of parental depression during adolescence with cognitive development in later life in China: A population-based cohort study
Source: PLoS Med. 2021 Jan 11;18(1):e1003464. doi: 10.1371/journal.pmed.1003464 (PMC7799791; doi:10.1371/journal.pmed.1003464)
Supplement: S4 Table — Models on the association of parental depression in 2012 with offspring cognitive test scores in the following years: (a) Mathematics and vocabulary test scores; (b) Immediate word recall, delayed word recall, and number series test scores. (DOCX) [file pmed.1003464.s005.docx]

**S4 Table. Models on the association of parental depression in 2012 with mathematics, vocabulary, immediate word-recall, delayed word-recall, and number series test scores**

1. **Mathematics and vocabulary test scores**

|  | **Mathematics** | |  | **Vocabulary** | |
| --- | --- | --- | --- | --- | --- |
|  | **2014 (N=1,672)** | **2018 (N=1,022)** |  | **2014 (N=1,692)** | **2018 (N=1,014)** |
| ***Adjusted for offspring characteristics*** |  |  |  |  |  |
| Maternal depression in 2012 | -1.033*** (-1.263, -0.802) | -0.750*** (-1.035, -0.465) |  | -1.556*** (-1.872, -1.239) | -3.451*** (-4.130, -2.771) |
| Paternal depression in 2012 | -1.528*** (-1.807, -1.248) | -1.416*** (-1.775, -1.056) |  | -0.871*** (-1.261, -0.482) | -4.621*** (-5.480, -3.762) |
| ***Adjusted for offspring and parents’ characteristics*** |  |  |  |  |  |
| Maternal depression in 2012 | -0.979*** (-1.213, -0.746) | -0.650** (-1.130, -0.171) |  | -1.592*** (-1.916, -1.268) | -2.279*** (-3.093, -1.466) |
| Paternal depression in 2012 | -1.411*** (-1.695, -1.126) | -1.862*** (-2.280, -1.443) |  | -1.106*** (-1.503, -0.710) | -3.952*** (-4.951, -2.952) |

1. **Immediate word-recall, delayed word-recall, and number series test scores**

|  | **Immediate word recall** | **Delayed word recall** | **Number sequence test** |
| --- | --- | --- | --- |
|  | **(N=1,343)** | **(N=1,244)** | **(N=1,354)** |
| ***Adjusted for offspring characteristics*** |  |  |  |
| Maternal depression in 2012 | -0.122** (-0.201, -0.042) | -0.132* (-0.247, -0.016) | -0.595*** (-0.798, -0.392) |
| Paternal depression in 2012 | -0.324*** (-0.418, -0.229) | -0.115 (-0.252, 0.021) | -1.580*** (-1.822, -1.338) |
| ***Adjusted for offspring and parents’ characteristics*** |  |  |  |
| Maternal depression in 2012 | -0.131** (-0.220, -0.042) | -0.270*** (-0.399, -0.142) | -0.527*** (-0.745, -0.309) |
| Paternal depression in 2012 | -0.330*** (-0.437, -0.222) | -0.196* (-0.350, -0.043) | -1.636*** (-1.899, -1.373) |

**Note:**

1. Offspring characteristics included offspring age, sex, and birth order. Parents’ characteristics included maternal and paternal education levels, mother’s age and father’s age, whether the offspring lived together with the mother, whether the offspring lived together with the father, father’s employment status, and mother’s employment status
2. *p<0.05, ** p<0.01, *** p<0.001
