# Supplementary material for: Associations of parental depression during adolescence with cognitive development in later life in China: A population-based cohort study
Source: PLoS Med. 2021 Jan 11;18(1):e1003464. doi: 10.1371/journal.pmed.1003464 (PMC7799791; doi:10.1371/journal.pmed.1003464)
Supplement: S7 Table — The association of parental depression in 2012 with offspring cognitive test scores in subsequent years, using multiple imputation to impute for observations loss of follow-up and observations with the values of key covariates missing: (a) Mathematics and vocabulary test scores in 2014 and 2018; (b) Immediate word recall, delayed word recall, and number series test scores in 2016. (DOCX) [file pmed.1003464.s008.docx]

**S7 Table. The association of parental depression in 2012 with offspring cognitive test scores in subsequent years, using multiple imputation to impute for observations loss of follow-up and observations with the values of key covariates missing**

1. **Mathematics and vocabulary test scores in 2014 & 2018**

|  | **Mathematics** | |  | **Vocabulary** | |
| --- | --- | --- | --- | --- | --- |
|  | **2014 (N=2,281)** | **2018 (N=2,281)** |  | **2014 (N=2,281)** | **2018 (N=2,281)** |
| ***Crude model*** |  |  |  |  |  |
| Maternal depression in 2012 | -0.933*** (-1.195, -0.671) | -0.734*** (-1.092, -0.375) |  | -1.471*** (-1.828, -1.113) | -2.595*** (-3.425, -1.764) |
| Paternal depression in 2012 | -1.408*** (-1.722, -1.095) | -1.163*** (-1.595, -0.730) |  | -1.046*** (-1.479, -0.614) | -4.225*** (-5.193, -3.256) |
| ***Fully-adjusted model*** |  |  |  |  |  |
| Maternal depression in 2012 | -0.501*** (-0.794, -0.207) | -0.215* (-0.383, -0.046) |  | -1.153*** (-1.535, -0.772) | -1.091* (-2.131, -0.052) |
| Paternal depression in 2012 | -0.847*** (-1.207, -0.488) | -1.128*** (-1.622, -0.633) |  | -0.673** (-1.134, -0.213) | -3.741*** (-4.950, -2.532) |

1. **Immediate word-recall, delayed word-recall, and number series test scores in 2016**

|  | **Immediate word recall** | **Delayed word recall** | **Number series test** |
| --- | --- | --- | --- |
|  | **(N=2,281)** | **(N=2,281)** | **(N=2,281)** |
| ***Crude model*** |  |  |  |
| Maternal depression in 2012 | -0.131** (-0.229, -0.033) | -0.102* (-0.205, 0.001) | -0.607*** (-0.854, -0.360) |
| Paternal depression in 2012 | -0.300*** (-0.416, -0.185) | -0.162** (-0.301, -0.023) | -1.377*** (-1.668, -1.086) |
| ***Fully-adjusted model*** |  |  |  |
| Maternal depression in 2012 | -0.167** (-0.279, -0.054) | -0.185* (-0.356, -0.013) | -0.380** (-0.658, -0.102) |
| Paternal depression in 2012 | -0.328*** (-0.466, -0.190) | -0.212* (-0.424, -0.001) | -1.232*** (-1.574, -0.891) |

**Note:**

1. *p<0.05, ** p<0.01, *** p<0.001
2. In the fully-adjusted models, we controlled for offspring (i.e., age, sex, and birth order), parents (i.e., maternal and paternal education levels, mother’s age and father’s age, whether the offspring lived together with the mother, whether the offspring lived together with the father, father’s employment status, and mother’s employment status) and household (i.e., place of residence, household income in log scale, and number of offspring in the household) characteristics.
