# Supplementary material for: Associations of parental depression during adolescence with cognitive development in later life in China: A population-based cohort study
Source: PLoS Med. 2021 Jan 11;18(1):e1003464. doi: 10.1371/journal.pmed.1003464 (PMC7799791; doi:10.1371/journal.pmed.1003464)
Supplement: S8 Table — The association of parental depression in 2012 with offspring cognitive test scores in subsequent years, controlling for child CES-D score in 2012: (a) Mathematics and vocabulary test scores in 2014 and 2018; (b) Immediate word recall, delayed word recall, and number series test scores in 2016. CES-D, Center for Epidemiologic Studies-Depression. (DOCX) [file pmed.1003464.s009.docx]

**S8 Table. The association of parental depression in 2012 with offspring cognitive test scores in subsequent years, controlling for child CES-D score in 2012**

1. **Mathematics and vocabulary test scores in 2014 & 2018**

|  | **Mathematics** | |  | **Vocabulary** | |
| --- | --- | --- | --- | --- | --- |
|  | **2014 (N=1,562)** | **2018 (N=931)** |  | **2014 (N=1,562)** | **2018 (N=1,315)** |
| Maternal depression in 2012 | -0.714*** (-0.966, -0.462) | -0.137* (-0.318, -0.044) |  | -1.401*** (-1.738, -1.064) | -1.727*** (-2.672, -0.782) |
| Paternal depression in 2012 | -1.172*** (-1.481, -0.863) | -1.844*** (-2.282, -1.406) |  | -0.843*** (-1.250, -0.437) | -4.234*** (-5.403, -3.065) |

1. **Immediate word-recall, delayed word-recall, and number series test scores in 2016**

|  | **Immediate word recall** | **Delayed word recall** | **Number series test** |
| --- | --- | --- | --- |
|  | **(N=1,217)** | **(N=1,179)** | **(N=1,225)** |
| Maternal depression in 2012 | -0.177*** (-0.271, -0.084) | -0.302*** (-0.441, -0.162) | -0.420*** (-0.650, -0.190) |
| Paternal depression in 2012 | -0.344*** (-0.459, -0.230) | -0.231* (-0.405, -0.058) | -1.551*** (-1.838, -1.265) |

**Note:**

1. *p<0.05, ** p<0.01, *** p<0.001
2. Besides child CES-D score in 2012, we controlled for offspring (i.e., age, sex, and birth order), parents (i.e., maternal and paternal education levels, mother’s age and father’s age, whether the offspring lived together with the mother, whether the offspring lived together with the father, father’s employment status, and mother’s employment status) and household (i.e., place of residence, household income in log scale, and number of offspring in the household) characteristics.
