# Supplementary material for: Associations of parental depression during adolescence with cognitive development in later life in China: A population-based cohort study
Source: PLoS Med. 2021 Jan 11;18(1):e1003464. doi: 10.1371/journal.pmed.1003464 (PMC7799791; doi:10.1371/journal.pmed.1003464)
Supplement: Attachment — Submitted filename: Response_to_Reviewers.docx [file pmed.1003464.s012.docx]

**Title:**

Original “**The effects of parental depression during adolescence on cognitive development in later life: a population based cohort study from China**”

Revised to “**Associations of parental depression during adolescence with cognitive development in later life, in China: a population-based cohort study**”

Manuscript ID: PMEDICINE-D-20-03333R1

Responses to Editors and Reviewers

Dear Editor,

Thank you for your comments as well as those from the reviewers.

We have provided point-by-point responses to your requests below and resubmitted a revised clean version. We have also tracked the changes in the file “Manuscript_tracked”.

We appreciate you reconsidering the manuscript and look forward to hearing from you in due course.

**Responses to the editor’s requests:**

1. Please address all of the reviewers’ comments below – further consideration of your manuscript will depend on satisfactory resolution of the concerns raised.

**Response:** In this document, we have included our point-by-point responses to all comments from the editor and the reviewers. We appreciate your reconsideration.

2. Title: Please update to: “Associations of parental depression during adolescence with cognitive development in later life, in China: a population based cohort study”

**Response:** Thanks for the suggestion. We have revised the title accordingly.

3. Abstract:
a. Please show age spread (eg. IQR) and % by sex.
b. Please list the main factors used for adjustment.
c. Please quantify the main results (with 95% CIs and p values).
d. In the last sentence of the Abstract Methods and Findings section, please describe the main limitation(s) of the study's methodology.

Response: We have revised the abstract accordingly. The revised “Methods and findings” section is as below, with changes underscored:

*Abstract: Methods and findings*

“This study used longitudinal data from the nationally representative China Family Panel Studies. The sample included 2,281 adolescents aged 10-15 years (the median age was 13 years with an interquartile range between 11 and 14 years) in 2012 when their parents were surveyed for depression symptoms with the 20-item Center for Epidemiological Studies-Depression (CES-D) scale. The sample was approximately balanced by gender, with 1,088 females (47.7%). We examined the associations of parental depression in 2012 with offspring cognitive performance (measured by mathematics, vocabulary, immediate word-recall, delayed word-recall, and number series tests) in subsequent years (i.e., 2014, 2016, 2018) using linear regression models, adjusting for various offspring (i.e., age, gender, birth order), parent (i.e., parents’ education level, age, whether living with the offspring, and employment status), and household characteristics (i.e., place of residence, household income, and the number of offspring). We found parental depression during adolescence to be significantly associated with worse cognitive performance in subsequent years, in both crude and adjusted models. For example, in the crude models, adolescents whose mothers had depression symptoms in 2012 scored 1.0 point lower (95% confidence interval [CI]: -1.2 to -0.8, p<.001) in mathematics in 2014 compared to those whose mothers did not have depression symptoms; after covariate adjustment, this difference marginally reduced to 0.8 points (95% CI: -1.0 to -0.5, p<.001); the associations between paternal depression and mathematical scores were also significant. These findings remained robust after adjusting for offspring earlier cognitive ability, offspring depression status, and parents’ cognitive ability. In line with the neuroplasticity theory, we observed stronger associations between maternal depression and mathematical and vocabulary scores among the younger adolescents (i.e., 10-11 years) than the older ones (i.e., 12-15 years). We also observed a stronger association of greater depression severity and worse mathematical scores. The primary limitations of this study were the relatively high attrition rate and residual confounding.”

4. Please avoid causal language throughout your manuscript (‘effect’, ‘affected’, etc.), since your study cannot prove causation, eg. “Parental depression during adolescence had adverse effects on offspring cognitive development…” (line 62); instead refer to ‘association’, eg. “Parental depression during adolescence was associated with adverse offspring cognitive development…”.

**Response:** We have checked throughout the manuscript and revised the causal language with more nuanced descriptions as you have indicated.

5. In the Funding Disclosure, please list the funders, or state “The author(s) received no specific funding for this work”.

**Response:** This work was supported by the Chinese Ministry of Education Humanities and Social Science Foundation (Grant number: 19YJC740058).

6. At this stage, we ask that you include a short, non-technical Author Summary of your research to make findings accessible to a wide audience that includes both scientists and non-scientists. The Author Summary should immediately follow the Abstract in your revised manuscript. This text is subject to editorial change and should be distinct from the scientific abstract. Please see our author guidelines for more information: <https://journals.plos.org/plosmedicine/s/revising-your-manuscript#loc-author-summary>

**Response:** We have added an “Author summary” section to the revised manuscript, following the guidelines provided by the website above. Please also see below:

*Author summary*

**Why was this study done?**

- Depression is the leading mental health related contributor to the Global Burden of Disease.
- A large body of research has underscored the negative association between parental depression in the perinatal period and offspring cognitive performance in early childhood.
- Little is known about the association between parental depression and offspring cognitive performance in adolescence, a critically sensitive period for neurodevelopment.

### What did the researchers do and find?

Using a nationally representative sample from China with 2,281 adolescents aged 10-15 years old in 2012, we explored the association between parental depression status and offspring cognitive performance (e.g., math, vocabulary, working memory) during adolescence over a period of 6 years.

We found that both maternal depression and paternal depression were associated with worse offspring cognitive performance up to six years later and across a range of cognitive performance tests, consistent with the theory emphasizing the importance of both parents in shaping cognitive outcomes during adolescence.

The associations remained robust even after adjusting for a wide range of confounding variables (e.g., gender, parents’ educational level, etc.), offspring historic cognitive performance in toddlerhood and in 2012, offspring depression status, and parents’ cognitive ability.

Stronger associations were found between maternal depression and younger adolescents’ cognitive performance (i.e., 10-11 years) than older ones (i.e., 12-15 years), which was aligned with neuroplasticity being more marked in younger adolescents.

### What do these findings mean?

- The inter-generational impact of parental depression on cognitive development, established for perinatal maternal depression in early childhood, extends well into adolescence when the associations are visible for both parents.
- This evidence furthers the understanding of the life course determinants of cognitive development and emphasizes the importance of preventing and treating parental depression beyond early childhood into adolescence.

7. Please use the "Vancouver" style for reference formatting, and see our website for other reference guidelines: https://journals.plos.org/plosmedicine/s/submission-guidelines#loc-references. Citations should be in square brackets, and preceding punctuation.

**Response:** We have revised the reference formatting using the “Vancouver” style and make sure to meet the reference guidelines for the journal.

8. Please correct “offsprings” to “offspring” throughout.
**Response:** We have changed “offsprings” to “offspring” throughout the manuscript.

9. Please avoid referring to non-significant results as “trend(s)”, eg. line 285; simply state whether or not the result is significant.

**Response:** We have removed the wording of “trend” and only described results as being significant or non-significant. Please see an example below. We have checked throughout the manuscript to avoid such ambiguous expression.

*Results: Severity of depression*

Original: “We observed clear trends for the hypothesized dose-response effect of maternal depression for all seven outcomes, with three associations reaching statistical significance (p<.05).”

Revised to: “We observed significant associations between the hypothesized dose-response effect of maternal depression and three cognitive outcomes, including delayed word recall, number sequence, and mathematics scores (Figure 3).”

10. Lines 364-368: Please remove the Funding and Competing Interests information, and instead include these in the online submission form.

**Response:** We have removed Funding and Competing Interests information from the main text, and will include them in the online submission form.

11. Lines 370-373: Please move the Ethics information to the Methods section.

**Response:** We have moved the Ethics information to the Methods section.

12. Did your study have a prospective protocol or analysis plan? Please state this (either way) early in the Methods section.
a) If a prospective analysis plan (from your funding proposal, IRB or other ethics committee submission, study protocol, or other planning document written before analyzing the data) was used in designing the study, please include the relevant prospectively written document with your revised manuscript as a Supporting Information file to be published alongside your study, and cite it in the Methods section. A legend for this file should be included at the end of your manuscript. 
b) If no such document exists, please make sure that the Methods section transparently describes when analyses were planned, and when/why any data-driven changes to analyses took place. 
c) In either case, changes in the analysis-- including those made in response to peer review comments-- should be identified as such in the Methods section of the paper, with rationale.

**Response:** We have submitted the prospective analysis plan as Supporting Information to the revised manuscript, and we also added the following description at the beginning of the Methods section:

*Methods* (para. 1):

“For the current study, the analysis plan was drafted in January 2020. During the revision process, in response to editors’ and reviewers’ comments, four sets of sensitivity analyses were added to address potential confounding issues and attribution bias (see analysis plan in the Supporting Information file).”

13. Please ensure that the study is reported according to the STROBE guideline, and include the completed STROBE checklist as Supporting Information. 1 Please add the following statement, or similar, to the Methods: "This study is reported as per the Strengthening the Reporting of Observational Studies in Epidemiology (STROBE) guideline (S1 Checklist)." The STROBE guideline can be found here: http://www.equator-network.org/reporting-guidelines/strobe/ When completing the checklist, please use section and paragraph numbers, rather than page numbers.

**Response:** We have included the completed STROBE checklist as Supporting Information. The following sentence was added to the Methods section:

*Methods* (para. 1)

“This study was reported as per the Strengthening the Reporting of Observational Studies in Epidemiology (STROBE) guideline ([S1 STROBE Checklist](https://journals.plos.org/plosmedicine/article?id=10.1371/journal.pmed.1003115#pmed.1003115.s001)).”

**Responses to Comments from Reviewer #1:**

**Reviewer #1:** I confine my remarks to statistical aspects of this paper. The general approach is fine, but I have two areas of concern to address before I can recommend publication.

First, the authors use causal language and this is not warranted. (Words such as "impact", "affect" and so on).

**Response:** We appreciate the reviewer’s comments. We have thoroughly revised the manuscript to avoid using language with potential causal inferences. Below are a few examples:

*Abstract: Conclusion*

Original: “Parental depression during adolescence had adverse effects on offspring cognitive development…”

Revised to: “Parental depression during adolescence was associated with adverse offspring cognitive development…”

*Introduction:*

Original: “We also aim to explore the duration of such effects...”

Revised to: “We also aim to explore the duration of such associations…”

*Discussion:*

Original: “we observed that boys and girls were equally affected by maternal depression…”

Revised to: “We observed that the cognitive performance of both boys and girls were equally associated with maternal depression…”

Second, the authors should not categorize continuous independent variables such as parent age and CESD scores. In *Regression Moideling Strategies* Frank Harrell lists 11 problems with this and sums up "nothing could be more disastrous". I wrote a blog post illustrating some problems with this graphically. <https://medium.com/@peterflom/what-happens-when-we-categorize-an-independent-variable-in-regression-77d4c5862b6c>

**Response:** We appreciate the reviewer’s comments.

We note that parental age was modelled as a continuous variable in the regression analysis. We only categorized age in the summary table and attrition table for easier presentation. We have revised the text to clarify this.

For the CES-D scores, we adhered to the norms used in previous studies [1, 2] wherein a cut-off score of 16 on the CES-D was used to classify persons as having clinical threshold depressive symptoms. This cutoff score has been validated with Diagnostic and Statistical Manual of Mental Disorders (DSM-III) criteria for clinical depression and has been used in diverse population [3-5]. Thus, we perceive it a validated approach to differentiating people with clinically significant depressive symptoms. In response to the reviewer’s comments, we also conducted a sensitivity analysis treating CES-D as a continuous variable (see **Appendix Table 6**). The results remained consistent. Relevant references have been inserted in the main text to testify our analytic approach.

**Responses to Comments from Review #2**

**Reviewer #2:** This manuscript reports the investigation of an important question about transgenerational effects of parental depression of offspring cognitive ability, and so the wider effects of parental depression on the life chances of the offspring. The sample is large and has many strengths, not least that it represents a population beyond the developed, Western world that contributes so much to the literature; the prospective design is appropriate and the analyses thoughtfully done. The manuscript is clear and the balance of reporting between the main text and supplementary material is good; the latter is necessary to support deeper reading.

For the editorial team the main question may be the extent to which this reports incremental advances by dint of the important sample, novel Chinese setting and methodologically elegant approach, or wehther it really breaks new ground. It is certainly a high quality paper. While the early developmental effects of parental (particularly maternal) depression on the child are well evidenced, later effects during puberty are less clear so this study is important. That said, the main paper illuninating this is pretty definitive, Shen et al., cited as #10, with a sample size of over a million and the ability to address the question left outstanding here as to whether the effects demonstrated have simply 'tracked' following a causally important effect earlier in life; there was also a well-cited commentary about the public health implications of their findings. This paper is cited appropriately and minor changes to the already excellent discussion to reflect what is definitive replication and what is advance could strengthen the work. Specifically, around line 308 I'd suggest the work contributes to preliminary evidence of causation, with the possibility having been around for some decades (eg the Murray, Cooper, Hay and other references).

**Responses:** We appreciate the reviewer’s encouraging comments. Following the reviewer’s suggestion, we have added a brief statement in the discussion section to highlight our potential contribution to the existing literature, including citations to some of the key papers by the authors listed by the reviewer). Below is the revised text:

*Discussion (Para. 2)*

“Our study addresses this knowledge gap using a nationally representative sample from China. We not only confirm the strong and consistent associations between both maternal and paternal depression and offspring cognitive development during adolescence, but also contribute to the growing evidence suggesting that these effects may be causal [6, 7].”

For the authors, I have the following additional suggestions, again mainly to do with the discussion and interpretation, especially around the preliminaries of judging causality in terms of bias and confounding. I've mentioned tracking over development of cause and effect that may have occurred during early life, or may have operated causally only during a later 'window'. Shen et al addressed this to a degree, and the 'washing out' of the effect in this current paper during adolescence may fit with this.

The effect sizes are small: around one point. These were robust to adjustment for sensible confounders but the danger of residual confounding is ever-present in this kind of developmental observational epidemiology. One offspring characteristic not measured (that might be seen as a mediator) is childhood depression, either through a genetic effect, or a transgenerational response to shared family or wider environment.

**Response:** We agree with the reviewers’ concerns regarding the possibility of residual confounding by factors operating in early life, notably childhood mental health status. That said, the specific variable mentioned, i.e. childhood depression is very rare, as depression typically emerges in adolescence and young adulthood [8]. In our study, we adjusted for multiple potential confounders through a serious of sensitivity analyses, including offspring depression status measured in 2012. In detail, we controlled for the following covariates respectively in the sensitivity analyses:

1) offspring cognitive performance during toddlerhood (i.e. age to speak a whole sentence, and age to count from 1 to 10);
2) offspring cognitive performance in 2012 when parental depression was assessed.
3) offspring CES-D score measured in 2012
4) parental cognitive scores measured in 2010.

The results of the sensitivity analyses were shown in **Table 3,** **Appendix Tables 8** and **9**, which were very consistent with the results in our main analyses. Nevertheless, we acknowledge that there remains the possibility of residual confounding and have revised the limitation part in the discussion section to further address this point, as below:

*Discussion (para. 5)*

“Fourth, we recognized that residual confounders, such as childhood depression, might have an impact on the association.”

On the small effect size, the fact that it appears more important for maths and vocab may be a function of the tests, themselves, given that these are much more granular (0-24 and 0-34) than for the other domains (0-10/15). I would make more of the consistent patterns and 'g' than the individual domains, but that's a contested area.

**Reponses**: We agree that the range of scale scores may influence the size of statistical estimates and have focused on the consistency of the association in our inferences.

The authors mention attrition as a possible contribution to the changes in effects over the follow-up (age effects). One approach could be to model this, perhaps through imputation. Again, a contested area but a systematic approach to imputing exposure, outcome and/or confounders would shed light on the bias and might be reassuring.

**Response:** We appreciate the reviewer’s comments and suggestions.

We argue that the attrition bias in the current study is likely to be small because there were no systematic differences in baseline offspring, parents, and household characteristics between participants followed-up and those who were lost (as shown in **Table 1** in our original manuscript).

Nevertheless, we conducted multiple imputation in a sensitivity analysis to address the potential attrition bias using the multiple imputation approach described in previous studies [9-11]. We imputed for observations loss of follow-up or for observations with key covariates missing in 2014, 2016, and 2018. In our imputation, we included all the variables on offspring characteristics, parents’ characteristics, household characteristics, as well as parental CES-D scores. The multiple imputation was carried out using the MI command in STATA 14.2. The results are shown in **Appendix Table 7**, which are very consistent with the main results. Relevant references for this methodology were inserted in the revised manuscript.

A small point: personally, I would be interested in the ethical pedigree of the data. The Harvard REC saw this as secondary analysis of anonymous data but what about the original collection? This is in no way meant to infer any problem; I am simply interested and believe it's a relevant aspect of describing the data in a global health setting and encouraging openness.

**Response:** The CFPS data was collected by the Institute of Social Science Survey of Peking University, China. All individuals signed personal consent forms and/or parental consent forms before participation [12]. The CFPS data is publicly accessible (with registration required) without any identifiable information about individuals. For example, to protect the individuals’ identity, the CFPS set restrictions on county-level data. Each county was assigned an anonymous ID, and all county-level data (e.g., each county’s GDP, per capita GDP, population, employment rate, etc) are blurred, with a random noise added to the actual values.

We slightly revised the ethical statement to better reflect the ethical review we have gone through, see the revised text below:

*Methods: Data source and study sample*

“This project used publicly-accessible secondary data obtained from the CFPS website (https://www.isss.pku.edu.cn/cfps/en/data/public/index.htm) which excludes all identifiable information about individual participants…”

**Responses to comments from Reviewer #3:**

**Reviewer #3:** This manuscript reports about an association between parental depression and children's cognitive development. There are several limitations.

Sampling.

The sampling is not clear. 16,000 households were sampled, but data were available for around 2000 households (?) in which depression data were available. It is important to know the difference between the panel study's sampling and available data. Were data constrained by parenting status, having children at home, refusal? More information is needed.

**Response:** We understand the reviewer’s comment and concern. There were around 16,000 households samples in the entire dataset. There were 2,406 offspring aged 10-15 years in the CFPS 2012 survey. We excluded 135 offspring with both parents’ depression measures marked as “unknown”, “refuse to answer”, or “inapplicable”. As such, the final target sample comprised 2,281 offspring. We have included this information in the “sample size” part in the Method section. Yet, we understand the author’s concern and have combined the original “data source” section and the “sample size” section to provide a more complete rationale for data selection. We also revised the language to better reflect why our final sample size is 2,281. The revised “Data source and sample size” is as below:

*Methods: Data source and the study sample*

“Data used in this study were from the China Family Panel Studies (CFPS). CFPS is a nationally representative, longitudinal survey conducted by the Institute of Social Science Survey at Peking University in collaboration with the Survey Research Center at the University of Michigan [12]. CFPS was first launched in 2010 with subsequent rounds of data collected in 2012, 2014, 2016, and 2018. The target sample of CFPS consists of around 16,000 Chinese households, representing 95% of the Chinese population [12]. CFPS collected information at the individual, household, and community levels on a range of topics, including parents’ physical and mental health, education and work status, economic activities, and offspring cognitive performance. Using a multistage probability-proportional-to-size sampling process, CFPS selected 144 counties from 25 provinces and 32 townships in Shanghai as the primary sampling unit. A total of 640 communities were randomly sampled within counties, and then 25 households were randomly selected from each community. All eligible households and household members were subjects of the survey[12].

Our study used the 2012 survey as the baseline when depression measurement for at least one parent was available. Of all the households included in the CFPS data, there were 2,406 offspring aged 10-15 years in the CFPS 2012 survey. Among them, we excluded 135 offspring with both parents’ depression measures marked as “unknown”, “refuse to answer”, or “inapplicable”. Thus, the final sample comprised 2,281 offspring.

To assess the impact of parental depression on offspring cognitive development, we set up the data in a panel form with parents’ depression status measured in 2012 and offspring cognitive performance measured in 2014, 2016, and 2018. Among the 2,281 offspring identified in 2012, we successfully followed up 85.9% of the offspring in 2014, 72.1% in 2016, and 63.7% in 2018 (**Table 1**). The number of offspring with valid cognitive measures also differed by survey waves. For example, among the 2,281 offspring, 320 of them were lost of follow-up in 2014, and another 250 of them had their vocabulary test score marked as “unknown” or “inapplicable”. As a result, in total, we included 1,711 offspring for the analysis on the vocabulary test in 2014. See **Figure 1** for details.”

The study analyzes longitudinal data, with a retention rate of about 67%. I am aware of the great difficulty of recruiting and maintaining samples intact (see the important National Academy Science (US) report on the topic.). Still, one wonders if sound epidemiology can really be done on samples with so much attrition. At minimum, the authors need to provide details about whether there are systematic sources of attrition, and to evaluate the bias introduced. Although the authors claim that there were not many differences, the reason for this is most likely because there was so much attrition. That is, attrition effects actually become harder to detect when attrition is high, and that's because one loses people from both sides of the distribution, but the difference in the 'risk side' is obscured. It would be useful to see a visual representation of who was lost, as a function of their characteristics.

**Response:** We appreciate the reviewer’s comments.

Among the 2,281 offspring identified in the 2012 survey, 1,960 (85.9%), 1,644 (72.1%), and 1,452 (63.7%) were followed-up in 2014, 2016, and 2018, respectively. Thus, our first follow-up had a very respectable completion rate, though this did fall with subsequent rounds (as might be expected). Following the widely adopted criteria in previous studies [13, 14], we determined that attrition bias in the current study is likely to be small because there were no systematic differences in baseline offspring, parents, and household characteristics between participants followed-up and those who were lost (as shown in **Table 1** in our original manuscript).

However, we acknowledge the reviewer’s concern and have now conducted a sensitivity analysis to address the attrition issue. Following the approach in earlier studies [9-11], we used multiple imputation to impute for observations loss of follow-up or for observations with key covariates missing in 2014, 2016, and 2018. In our imputation, we included all the variables on offspring characteristics, parents’ characteristics, household characteristics, as well as parental CES-D scores. The results are shown in **Appendix Table 7**, which are very consistent with the main results. Relevant parts were revised in the revised manuscript, for instance:

*Methods (Statistical analysis):*

“4) We adopted multiple imputation to impute for observations loss of follow-up or for observations with key covariates missing in 2014, 2016, and 2018. The multiple imputation was carried out using MI command in STATA. In our imputation, we included all the variables on offspring characteristics, parents’ characteristics, and household characteristics, as well as parental CES-D scores.”

*Results (Sensitivity analyses):*

In the fourth sensitivity analysis, we imputed the test scores and other key covariates for individuals loss of follow-up over years or with no valid data. The results remained consistent (**Appendix Table 7**).

Measurement.

The authors focus on depression, using the CES-D instrument. This is a widely used instrument. But, does it measure depression per se, or does it simply capture psychological distress more broadly? There is good reason to believe the latter. Moreover, simply because one administers a single mental health measure, it is not the case that the measure actually assesses that construct alone; in fact, the measure most likely picks up comorbid conditions. The reason this point is important is because the interpretive framework for this paper is depression, but there is way to establish that depression per, and not mental distress more generally, is being assessed.  

**Response:** We agree with the reviewer that depression is often co-morbid with anxiety and that, together, they do constitute “psychological distress”. That said, there are also specificities which differentiate these two domains of psychopathology, particularly in the context of our study: thus, while there is strong evidence of the association between maternal depression (typically measured using symptom severity scales such as the CES-D) and child outcomes, there is no such association observed for maternal anxiety. Arguably, the CES-D is one of the most widespread, economical, and validated scales for assessing depression in population-based studies [1, 3, 15]. A recent meta-analysis reviewed 28 studies (10,617 participants) that used CES-D as the screening instrument to measure depression and concluded that it has acceptable screening accuracy (i.e. criterion validity) in the general population or primary care settings [16].

The authors note the scoring guidelines come from the American Psychological Association. That's not quite correct. The APA does have a link which describes the instrument, but that is not an APA guideline or endorsement; it is simply a description of the measure.

**Response:** We apologize for the mis-interpretation of the information source. We have corrected the statement in the revised manuscript. Please see revised texts below:

*Methods: Exposure measure*

“Following the traditional standard proposed by the scale developer [2] and evidence from validation studies [3-5], we defined parents scoring 16 or higher on CES-D 20 as the ones with depression symptoms…”

Covariates.

The most important variable that is necessary to aid interpretation of the results would be the cognitive status of the parents. This is, are we observing the putative effects of depression or are we observing the intergenerational transmission of cognitive ability (which happens to also be correlated with depression). Education is a fine covariate, but it is not an entirely adequate proxy for cognitive ability.

**Response:** Parents’ educational level is the most widely used proxy indicator of parental cognitive ability, and the positive association between the two has been consistently documented in previous studies [17, 18], and particularly in the Chinese population context [19, 20].

To further address this concern, we conducted a sensitivity analysis adding parental cognitive scores (i.e., math test and word test scores) measured in 2010 to the fully-adjusted models to control for the effects of transgenerational transmission of cognitive ability. The results are shown in **Appendix Table 9.** The results are consistent with in the main analysis, except that the association between maternal depression and number series test was no longer statistically significant.

Analysis. 

The effect modification analysis has yielded several interaction terms. Given the multiple test performed, these need to corrected for multiple testing. In addition, in performing these effect modification analyses, the authors need to estimate a fully saturated model, not simply one in which covariates are entered as main effects. There is actually a fairly sizeable literature on this topic, and not all of it says the same thing, but the fully saturated model should evaluated to see how it affects effect modification parameters.

**Response:** In the current study, effect modification analyses only include one interaction term at a time. For example, when investigating the effect modification by offspring gender, we only added the interaction term between offspring gender and maternal/paternal depression status to the fully-adjusted regression. Moreover, all of our effect modification analyses were based on fully saturated model. That is to say, we adjusted all offspring, parents, and household characteristics. We have revised our text to clarify these points, see below:

*Methods: Statistical analysis*

**“**First, we explored the effect modification by offspring age at exposure, using fully-adjusted linear regression models with the interaction term between offspring age and maternal/paternal depression status. Then, we used the fully-adjusted model with interaction term between offspring gender and maternal/paternal depression status to examine the effect modification by offspring gender.**”**

Interpretation.

Given the above, especially the absence of a cognitive covariate from the parental generation and broader mental health assessment, it is not possible to interpret the results in terms of possible depression effects of cognitive development.

**Response:** As indicated in our responses above, a sensitivity analysis with parental cognitive scores as a control variable show consistent results with our original analysis. Meanwhile, CES-D has been widely used as a validated measure for depression in the general population. Therefore, we believe our findings offer strong evidence of a consistent association between parental depression and offspring cognitive development in adolescence, after accounting for a range of confounding variables. However, we understand the reviewer’s concern and have revised the writing with more cautious interpretations, for instance, by replacing causal wording with more nuanced language (e.g., effects 🡪 associations).

Editing.

A bit more attention to writing is needed, with occasional grammatical lapses. 
"Most research on the effects of parental depression on offspring cognitive outcomes have been limited…"
"the associations were temporal such that parental parental depression diagnosed during adolescence were…"

**Response:** We appreciate the reviewer’s careful reading of our paper. We have edited the problematic sentences and thoroughly proofread the entire manuscript to ensure grammatical accuracy.

**References**

1.	Andresen EM, Malmgren JA, Carter WB, Patrick DL. Screening for depression in well older adults: Evaluation of a short form of the CES-D. American journal of preventive medicine. 1994;10(2):77-84.

2.	Radloff LS. A self-report depression scale for research in the general population. Applied psychol Measurements. 1977;1:385-401.

3.	Weissman MM, Sholomskas D, Pottenger M, Prusoff BA, Locke BZ. Assessing depressive symptoms in five psychiatric populations: a validation study. American journal of epidemiology. 1977;106(3):203-14.

4.	Li Z, Hicks MH-R. The CES-D in Chinese American women: Construct validity, diagnostic validity for major depression, and cultural response bias. Psychiatry research. 2010;175(3):227-32.

5.	Stahl D, Sum CF, Lum SS, Liow PH, Chan YH, Verma S, et al. Screening for depressive symptoms: validation of the center for epidemiologic studies depression scale (CES-D) in a multiethnic group of patients with diabetes in Singapore. Diabetes Care. 2008;31(6):1118-9.

6.	Murray L, Arteche A, Fearon P, Halligan S, Croudace T, Cooper P. The effects of maternal postnatal depression and child sex on academic performance at age 16 years: a developmental approach. Journal of child Psychology and Psychiatry. 2010;51(10):1150-9.

7.	Hay DF, Asten P, Mills A, Kumar R, Pawlby S, Sharp D. Intellectual problems shown by 11-year-old children whose mothers had postnatal depression. The Journal of Child Psychology and Psychiatry and Allied Disciplines. 2001;42(7):871-89.

8.	Patel V, Flisher AJ, Hetrick S, McGorry P. Mental health of young people: a global public-health challenge. The Lancet. 2007;369(9569):1302-13.

9.	Asendorpf JB, Van De Schoot R, Denissen JJ, Hutteman R. Reducing bias due to systematic attrition in longitudinal studies: The benefits of multiple imputation. International Journal of Behavioral Development. 2014;38(5):453-60.

10.	Biering K, Hjollund NH, Frydenberg M. Using multiple imputation to deal with missing data and attrition in longitudinal studies with repeated measures of patient-reported outcomes. Clinical epidemiology. 2015;7:91.

11.	Twisk J, de Vente W. Attrition in longitudinal studies: how to deal with missing data. Journal of clinical epidemiology. 2002;55(4):329-37.

12.	Xie Y, Hu J. An introduction to the China family panel studies (CFPS). Chinese Sociological Review. 2014;47(1):3-29.

13.	Miller RB, Wright DW. Detecting and correcting attrition bias in longitudinal family research. Journal of Marriage and the Family. 1995:921-9.

14.	Morrison TC, Wahlgren DR, Hovell MF, Zakarian J, Burkham-Kreitner S, Hofstetter CR, et al. Tracking and follow-up of 16,915 adolescents: minimizing attrition bias. Controlled clinical trials. 1997;18(5):383-96.

15.	Kessler RC, Andrews G, Colpe LJ, Hiripi E, Mroczek DK, Normand S-L, et al. Short screening scales to monitor population prevalences and trends in non-specific psychological distress. Psychological medicine. 2002;32(6):959-76.

16.	Vilagut G, Forero CG, Barbaglia G, Alonso J. Screening for depression in the general population with the Center for Epidemiologic Studies Depression (CES-D): a systematic review with meta-analysis. PloS one. 2016;11(5):e0155431.

17.	Heckman J, Vytlacil E. Identifying the role of cognitive ability in explaining the level of and change in the return to schooling. Review of Economics and Statistics. 2001;83(1):1-12.

18.	Hatch SL, Feinstein L, Link BG, Wadsworth ME, Richards M. The continuing benefits of education: adult education and midlife cognitive ability in the British 1946 birth cohort. The Journals of Gerontology Series B: Psychological Sciences and Social Sciences. 2007;62(6):S404-S14.

19.	Huang G, Xie Y, Xu H. Cognitive ability: Social correlates and consequences in contemporary China. Chinese Sociological Review. 2015;47(4):287-313.

20.	Liu C, Luo R, Yi H, Zhang L, Li S, Bai Y, et al. Soil-transmitted helminths in southwestern China: a cross-sectional study of links to cognitive ability, nutrition, and school performance among children. PLoS neglected tropical diseases. 2015;9(6):e0003877.
