# Supplementary material for: Associations of parental depression during adolescence with cognitive development in later life in China: A population-based cohort study
Source: PLoS Med. 2021 Jan 11;18(1):e1003464. doi: 10.1371/journal.pmed.1003464 (PMC7799791; doi:10.1371/journal.pmed.1003464)
Supplement: Attachment — Submitted filename: Response_to_Reviewers.docx [file pmed.1003464.s013.docx]

**Title:**

Original “**The effects of parental depression during adolescence on cognitive development in later life: a population based cohort study from China**”

Revised to “**Associations of parental depression during adolescence with cognitive development in later life, in China: a population-based cohort study**”

Manuscript ID: PMEDICINE-D-20-03333R2

Responses to Editors and Reviewers

Dear Editor,

Thank you for your comments as well as those from the reviewers.

We have provided point-by-point responses to your requests below and resubmitted a revised clean version. We have also tracked the changes in the file “Manuscript_tracked”.

We appreciate you reconsidering the manuscript and look forward to hearing from you in due course.

**Responses to the editor’s requests:**

1.	Competing Interests: Please update the relevant sentence to our standard text: “VP is a member of PLOS Medicine's editorial board."

**Response:** We updated the competing interests accordingly in the submission system.

2.	Please provide line numbers in the margin throughout.

**Response:** We added line numbers in the margin throughout.

3.	Please give p values as “p<0.001” throughout, instead of “p<.001”. Please give exact values where greater than 0.001, including in Tables. In the Supplementary Information Tables, please do not report p=0.000; report as p<0.001.

**Response:** We have revised p value to “p<0.001” and showed the exact p values greater than 0.001. In the Supplementary information tables, we changed p=0.000 to p<0.001.

4.	The terms gender and sex are not interchangeable (as discussed in <http://www.who.int/gender/whatisgender/en/> ); please use the appropriate term throughout.
**Response:** We have change the term to “sex” throughout the manuscript.

5.	Abstract:
a.	Please quantify the main results (with 95% CIs and p values), including the following sentences:
i.	“the associations between paternal depression and mathematical scores were also significant.”
ii.	“These findings remained robust after adjusting for offspring earlier cognitive ability, offspring depression status, and parents’ cognitive ability.”
iii.	“In line with the neuroplasticity theory, we observed stronger associations between maternal depression and mathematical / vocabulary scores among the younger adolescents (i.e., 10-11 years) than the older ones (i.e., 12-15 years).”

**Response**: We have quantified the main results in the abstract. We showed some examples as below:

“For example, in the crude models, adolescents whose mothers had depression symptoms in 2012 scored 1.0 point lower (95% confidence interval [CI]: -1.2 to -0.8, p<0.001) in mathematics in 2014 compared to those whose mothers did not have depression symptoms; after covariate adjustment, this difference marginally reduced to 0.8 points (95% CI: -1.0 to -0.5, p<0.001); the associations remained robust after further adjusting for offspring earlier cognitive ability in toddlerhood (-1.8, 95% CI: -2.1, -1.4, p<0.001), offspring cognitive ability in 2012 (-0.9, 95% CI: -1.2, -0.6, p<0.001), offspring depression status (-0.7, 95% CI: -1.0, -0.5, p<0.001) , and parents’ cognitive ability (-0.8, 95% CI: -1.2, -0.3, p<0.001).”

“In line with the neuroplasticity theory, we observed stronger associations between maternal depression and mathematical/vocabulary scores among the younger adolescents (i.e., 10-11 years) than the older ones (i.e., 12-15 years). For example, the association between maternal depression and 2014 vocabulary scores was estimated to be -2.1 (95% CI: -2.6, -1.6, p<0.001) in those aged 10-11 years, compared to -1.2 (95% CI: -1.6, -0.8, p<0.001) in those aged 12-15 years with a difference of 0.9 (95% CI: 0.2, 1.6, p=0.010).”

b.	Conclusion: Begin with “In this study, we observed that…”
**Response**: We have revised the conclusion to start with “In this study, we observed that parental depression during adolescence…”

6.	Introduction: We recommend you delete this sentence, or move it to the Discussion: “If our hypotheses proved to be correct, our findings would point to the importance of addressing parental depression in the context of cognitive development and educational attainment well beyond childhood.”

**Response**: We deleted the sentence from the introduction and relevant implications were included in the discussion..

7.	Throughout, please give ages as “aged x years”, rather than “aged x years old”.
**Response**: We have changed “aged x years old” to “aged x years” throughout the manuscript.

8.	Methods: Please mention that your study did not require additional specific approval from an ethics committee.
**Response**: We added the sentence below to the method section:

“This study did not require additional specific approval from an ethics committee.”

9.	Results: Please refer to ‘association’ instead of ‘effect’ here and elsewhere: “For example, the effect of maternal depression on vocabulary scores in 2014 was -2.1 (95% CI: -2.6, -1.5) in those aged 10-11 years, comparing to -1.2 (95% CI: -1.6, -0.8) in those aged 12-15 years. Similarly, the effect of paternal depression on 2014 vocabulary scores was -1.7 (95% CI: -2.4, -1.0) in those aged 10-11 years, compared to -0.7 (95% CI: -1.1, -0.2) in those aged 12-15 years.”

**Response**: We have changed “effect” to “association” throughout the manuscript.

10.	Discussion: Please refer to ‘association’ instead of ‘effect’ here and elsewhere: “In our study, we observed that the cognitive performance of both boys and girls were equally associated with maternal depression, whereas the effects of paternal depression on boys and girls appeared more variable.”

**Response**: We have changed “effect” to “association” throughout the manuscript.

11.	Please remove spaces from within citation callouts throughout, eg “…hinder early childhood cognitive development [1,2].”

**Response**: We have removed all spaces from within citation callouts.

12.	Please replace hyperlinks here with citations: “…guidance online regarding when an IRB application is required and the IRB Decision Tool.”

**Response**: We have replaced the hyperlink with a citation of the IRB website.

13.	Results: Please quantify all comparisons with p values, as well as 95% CIs.

**Response**: We have quantified all comparisons with p values and 95% CIs. Several examples are showing below:

“For example, the association between maternal depression and mathematical scores in 2014 was estimated to be -1.1 (95% CI: -1.5, -0.7, p<0.001) in those aged 10-11 years, comparing to -0.5 (95% CI: -0.8, -0.2, p=0.001) in those aged 12-15 years; the difference between the two age groups was 0.6 (95% CI: 0.2, 1.1, p=0.006)…”

“For example, paternal depression was associated with 0.6 points lower (95% CI: -0.7, -0.4, p<0.001) immediate word-recall scores in 2016 in girls, but not in boys (95% CI: -0.2, 0.1, p=0.423); the difference was estimated to be 0.6 points (95% CI: 0.3,0.9, p<0.001).”

“…the association between maternal depression and vocabulary scores in 2014 was -2.1 (95% CI: -2.6, -1.6, p<0.001) in those aged 10-11 years, significantly lower than those aged 12-15 years (-1.2, 95% CI: -1.6, -0.8, p<0.001) with a difference of 0.9 (95% CI: 0.2, 1.6, p=0.010)”

14.	Tables: Where presenting data in Chinese Yuan, please also include an estimate in a more widely circulating currency, eg. USD.
**Response**: We have replaced the presentation in Chinese Yuan to USD.

15.	Please upload the analysis plan and STROBE checklist as separate files, with the names “S1 Text” and “S1 Checklist”, respectively.
**Response**: We have uploaded the files following this guideline.

16.	Please provide volume/issue/page or DOI for reference 35.

**Response**: Reference information has been added.

**Responses to Comments from Reviewer #1:**

Reviewer #1: The authors have addressed my concerns and I now recommend publication. 

Peter Flom

**Response**: We appreciate the reviewer’s compliment.

Reviewer #2: The authors have done a good job. Thank you for doing the additional analysis regarding attrition, for illuminating the ethics pedigree, and for the other changes.

Expunging the causal language is a great improvement, but 'The inter-generational impact of parental depression on cognitive development, established for perinatal maternal depression in early childhood, extends well into adolescence when the associations are visible for both parents.' is still pretty strong for an observational study (there will be residual confounding!). Perhaps 'Our results are consistent with an inter-generational etc.' or something a little less excited and in tune with the excellent discussion.

**Response:** We appreciate the reviewer’s suggestion to further refine the causal language. We have revised this sentence to below:

“Our results are consistent with the inter-generational association between parental depression and offspring cognitive development established in early childhood, but extend such association well into adolescence when the associations are visible for both parents. ”

Reviewer #3: This is a responsive revision, for the most part. 

On the whole, I am underwhelmed by the research.  I am not certain that it is especially novel.  The one thing that makes it stand out is that it is non-Western.  But, that's really it. 

**Response:** We appreciate the reviewer’s comments. The novelity of this study has been thoroughly summarized in the last paragraph of the discussion section. We believe the study, despite of its limitation, could make great contribution to the field by extending a significant and prevalent topic to an understudied population (e.g., a non-Western developing country) and investigating a critical age period of cognitive development (adolescence).

A few outstanding items. 

The authors did not appear to understand my comment about fully-saturated models.  Some statisticians argue that in order to interpret interaction effects, it is not enough to enter covariates, but one must enter ALL the interaction terms, including those with the covariates, into the models.  The authors have only entered covariates as main effects, not as part of interaction terms.  They should examine the implications of doing so.  You might want to seek statistical advice on this score.  
**Response:** We thank the reviewer’s suggestion. The statistical method we adopted has been widely used in other high-level publications on similar research area. We selected a couple of them and listed as below.

- Shen H, Magnusson C, Rai D, Lundberg M, Le-Scherban F, Dalman C, et al. Associations of parental depression with child school performance at age 16 years in Sweden. JAMA Psychiatry. 2016;73(3):239-46.
- Verkuijl NE, Richter L, Norris SA, Stein A, Avan B, Ramchandani PG. Postnatal depressive symptoms and child psychological development at 10 years: a prospective study of longitudinal data from the South African Birth to Twenty cohort. The Lancet Psychiatry. 2014;1(6):454-60.
- Netsi E, Pearson RM, Murray L, Cooper P, Craske MG, Stein A. Association of persistent and severe postnatal depression with child outcomes. JAMA psychiatry. 2018 Mar 1;75(3):247-53.

The fully saturated model is rarely used in studies of this area because it will cause substantial lose of power with multiple interaction terms, especially given the moderate sample size we have. We also note that the statistical reviewer has confirmed the appropriateness of our methods and models.

The authors also did not understand my comment about comorbidity.  Depression is not only comorbid with anxiety.  It is comorbid with many conditions.  For example, children who meet diagnostic criteria for conduct disorder are at heightened risk of depression.  As such, I do not believe that one can actually speak of depression 'effects' in the absence of information about the many conditions which depression is correlated.  This particular measure of depression, which taps into negative emotionality, is part and parcel of multiple psychiatric conditions.

**Response:** We have provided a thorough explanation on this issue in our previous response. However, we understand the reviewer’s concern and thus we have added the point as a potential limitation as below:

“Lastly, we cannot fully exclude comorbidity. It is possible that what we captured was actually the comorbidity of depression and other mental or physical illnesses.”
